# Supplementary figures and images for: Genome-wide analysis of Schistosoma mansoni reveals limited population structure and possible praziquantel drug selection pressure within Ugandan hot-spot communities
Source: PLoS Negl Trop Dis. 2022 Aug 18;16(8):e0010188. doi: 10.1371/journal.pntd.0010188 (PMC9426917; doi:10.1371/journal.pntd.0010188)

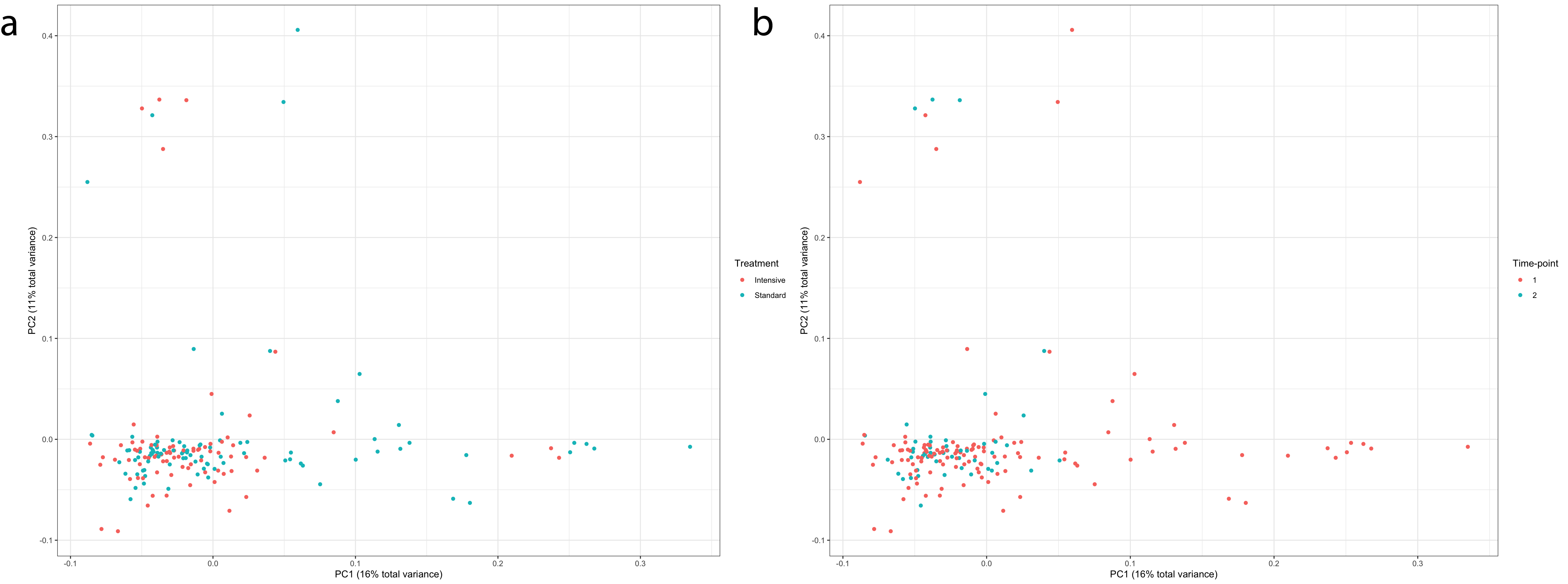

Supplement: S2 Fig — (TIF) [file pntd.0010188.s002.tif]

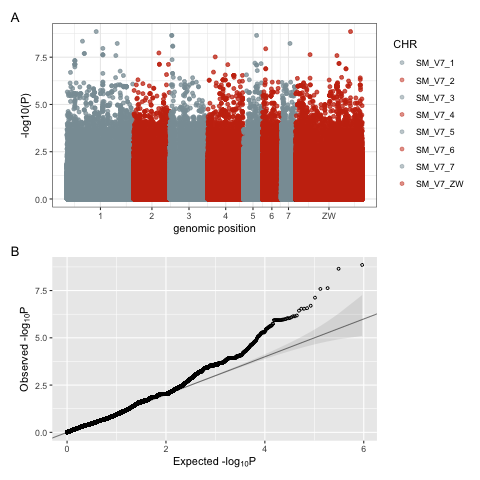

Supplement: S3 Fig — (A) Manhattan plot of unadjusted -log10 p-values for association of individual SNPs with per-individual mean egg-reduction rates. (B) QQplot of p-values from the same analysis against expectations under the null hypothesis. (TIFF) [file pntd.0010188.s003.tiff]

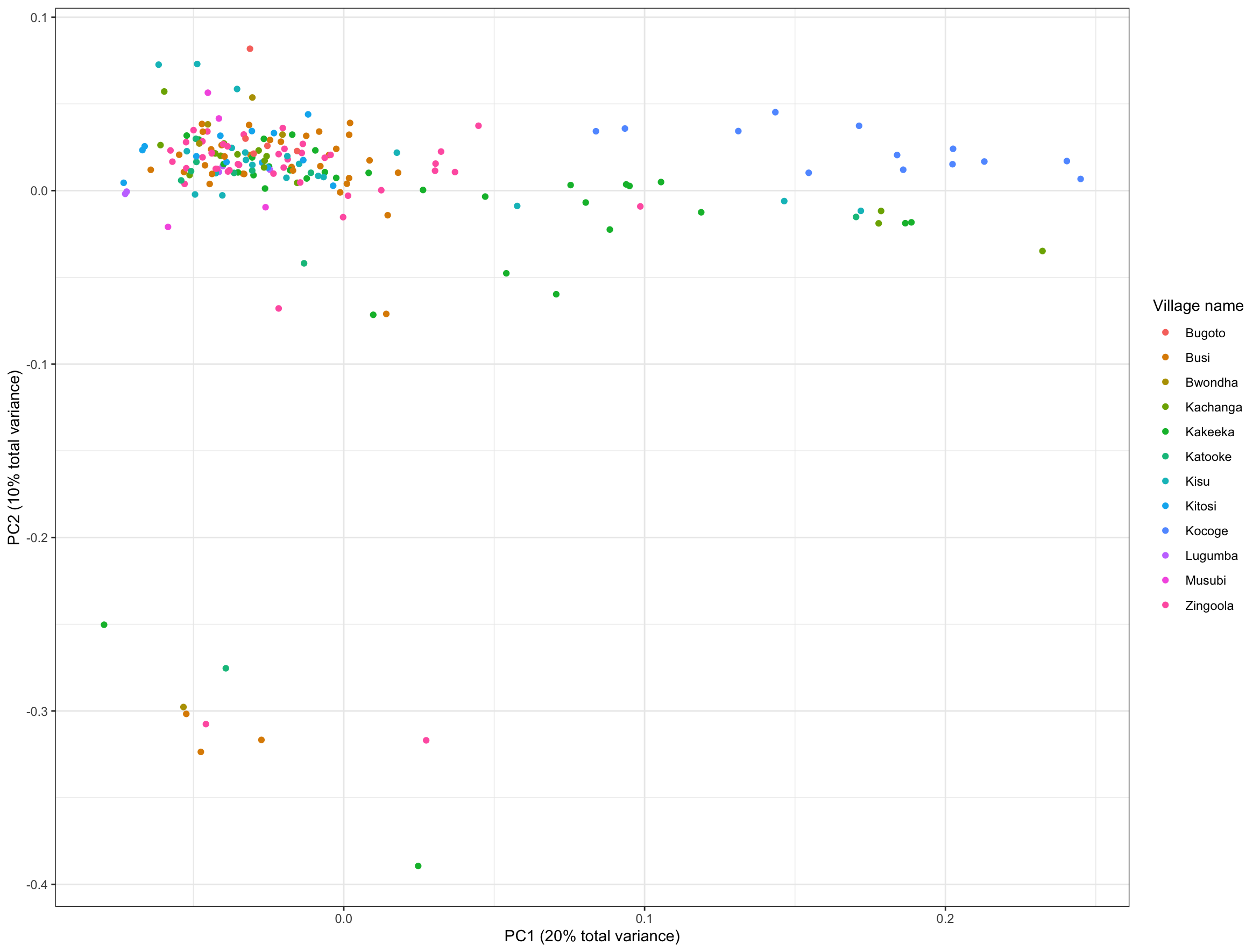

Supplement: S4 Fig — (TIFF) [file pntd.0010188.s004.tiff]
