# Supplementary material for: Genome-wide analysis of Schistosoma mansoni reveals limited population structure and possible praziquantel drug selection pressure within Ugandan hot-spot communities
Source: PLoS Negl Trop Dis. 2022 Aug 18;16(8):e0010188. doi: 10.1371/journal.pntd.0010188 (PMC9426917; doi:10.1371/journal.pntd.0010188)
Supplement: S1 Table — (DOCX) [file pntd.0010188.s005.docx]

**S1 Table.** Accession numbers and metadata for all samples included in analyses.

| **sample_ID** | **collection_date** | **village** | **treatment_arm** | **island** | **accession_** | **pre_post** | **patient** |
| --- | --- | --- | --- | --- | --- | --- | --- |
| 5582STDY7724293 | 10/08/2017 | Kachanga | Standard | Damba | ERS2891555 | pre_treatment | 75 |
| 5582STDY7759949 | 26/09/2017 | Zingoola | Standard | Koome | ERS2983612 | post_treatment | 46 |
| 5582STDY7724231 | 23/08/2017 | Zingoola | Standard | Koome | ERS2891493 | pre_treatment | 46 |
| 5582STDY7724309 | 10/08/2017 | Kachanga | Standard | Damba | ERS2891571 | pre_treatment | 76 |
| 5582STDY7724319 | 15/11/2017 | Kakeeka | Standard | Damba | ERS2891581 | post_treatment | 20 |
| 5582STDY7770933 | 10/07/2017 | Katooke | Intensive | Damba | ERS3016692 | pre_treatment | 68 |
| 5582STDY7770941 | 11/07/2017 | Katooke | Intensive | Damba | ERS3016700 | pre_treatment | 68 |
| 5582STDY7771009 | 10/07/2017 | Katooke | Intensive | Damba | ERS3016761 | pre_treatment | 68 |
| 5582STDY7770939 | 18/10/2017 | Busi | Intensive | Koome | ERS3016698 | pre_treatment | 1 |
| 5582STDY7724259 | 18/10/2017 | Busi | Intensive | Koome | ERS2891521 | pre_treatment | 1 |
| 5582STDY7724252 | 22/11/2017 | Busi | Intensive | Koome | ERS2891514 | post_treatment | 1 |
| 5582STDY7770956 | 17/10/2017 | Busi | Intensive | Koome | ERS3016715 | pre_treatment | 2 |
| 5582STDY7724233 | 18/10/2017 | Busi | Intensive | Koome | ERS2891495 | pre_treatment | 2 |
| 5582STDY7724277 | 23/11/2017 | Busi | Intensive | Koome | ERS2891539 | post_treatment | 3 |
| 5582STDY7724243 | 18/10/2017 | Busi | Intensive | Koome | ERS2891505 | pre_treatment | 3 |
| 5582STDY7724260 | 22/11/2017 | Busi | Intensive | Koome | ERS2891522 | post_treatment | 3 |
| 5582STDY7770991 | 19/10/2017 | Busi | Intensive | Koome | ERS3016764 | pre_treatment | 4 |
| 5582STDY7724269 | 21/11/2017 | Busi | Intensive | Koome | ERS2891531 | post_treatment | 4 |
| 5582STDY7770964 | 10/10/2017 | Kakeeka | Standard | Damba | ERS3016723 | pre_treatment | 21 |
| 5582STDY7771101 | 11/10/2017 | Kakeeka | Standard | Damba | ERS3016807 | pre_treatment | 22 |
| 5582STDY7724274 | 11/10/2017 | Kakeeka | Standard | Damba | ERS2891536 | pre_treatment | 22 |
| 5582STDY7724320 | 14/11/2017 | Kakeeka | Standard | Damba | ERS2891582 | post_treatment | 22 |
| 5582STDY7771003 | 11/10/2017 | Kakeeka | Standard | Damba | ERS3016755 | pre_treatment | 23 |
| 5582STDY7724265 | 10/10/2017 | Kakeeka | Standard | Damba | ERS2891527 | pre_treatment | 23 |
| 5582STDY7724281 | 09/10/2017 | Kakeeka | Standard | Damba | ERS2891543 | pre_treatment | 23 |
| 5582STDY7724240 | 13/11/2017 | Kakeeka | Standard | Damba | ERS2891502 | post_treatment | 23 |
| 5582STDY7771099 | 22/08/2017 | Zingoola | Standard | Koome | ERS3016805 | pre_treatment | 47 |
| 5582STDY7771043 | 23/08/2017 | Zingoola | Standard | Koome | ERS3016784 | pre_treatment | 48 |
| 5582STDY7724322 | 24/08/2017 | Zingoola | Standard | Koome | ERS2891584 | pre_treatment | 48 |
| 5582STDY7770915 | 23/08/2017 | Zingoola | Standard | Koome | ERS3016674 | pre_treatment | 48 |
| 5582STDY7724285 | 09/08/2017 | Kachanga | Standard | Damba | ERS2891547 | pre_treatment | 77 |
| 5582STDY7724288 | 07/11/2017 | Lugumba | Standard | Lugumba | ERS2891550 | pre_treatment | 94 |
| 5582STDY7724312 | 07/11/2017 | Lugumba | Standard | Lugumba | ERS2891574 | pre_treatment | 94 |
| 5582STDY7770937 | 13/09/2017 | Kachanga | Standard | Damba | ERS3016696 | post_treatment | 78 |
| 5582STDY7724250 | 09/10/2017 | Kakeeka | Standard | Damba | ERS2891512 | pre_treatment | 24 |
| 5582STDY7771004 | 11/09/2017 | Kachanga | Standard | Damba | ERS3016756 | post_treatment | 79 |
| 5582STDY7759911 | 24/10/2017 | Kitosi | Intensive | Koome | ERS2983579 | pre_treatment | 87 |
| 5582STDY7759950 | 23/10/2017 | Kitosi | Intensive | Koome | ERS2983613 | pre_treatment | 87 |
| 5582STDY7759951 | 22/11/2017 | Kitosi | Intensive | Koome | ERS2983614 | post_treatment | 87 |
| 5582STDY7759895 | 25/10/2017 | Kitosi | Intensive | Koome | ERS2983558 | pre_treatment | 87 |
| 5582STDY7759975 | 27/11/2017 | Kitosi | Intensive | Koome | ERS2983632 | post_treatment | 88 |
| 5582STDY7770926 | 10/07/2017 | Katooke | Intensive | Damba | ERS3016685 | pre_treatment | 69 |
| 5582STDY7759885 | 27/09/2017 | Zingoola | Standard | Koome | ERS2983563 | post_treatment | 49 |
| 5582STDY7770928 | 27/09/2017 | Zingoola | Standard | Koome | ERS3016687 | post_treatment | 49 |
| 5582STDY7770942 | 09/10/2017 | Kakeeka | Standard | Damba | ERS3016701 | pre_treatment | 25 |
| 5582STDY7770948 | 11/10/2017 | Kakeeka | Standard | Damba | ERS3016707 | pre_treatment | 25 |
| 5582STDY7771093 | 10/10/2017 | Kakeeka | Standard | Damba | ERS3016804 | pre_treatment | 25 |
| 5582STDY7724242 | 09/10/2017 | Kakeeka | Standard | Damba | ERS2891504 | pre_treatment | 25 |
| 5582STDY7724297 | 11/10/2017 | Kakeeka | Standard | Damba | ERS2891559 | pre_treatment | 26 |
| 5582STDY7771022 | 10/10/2017 | Kakeeka | Standard | Damba | ERS3016777 | pre_treatment | 26 |
| 5582STDY7770987 | 12/10/2017 | Kakeeka | Standard | Damba | ERS3016746 | pre_treatment | 27 |
| 5582STDY7771035 | 23/08/2017 | Zingoola | Standard | Koome | ERS3016781 | pre_treatment | 50 |
| 5582STDY7759963 | 31/10/2017 | Kisu | Intensive | Damba | ERS2983622 | pre_treatment | 36 |
| 5582STDY7770938 | 06/12/2017 | Kisu | Intensive | Damba | ERS3016697 | post_treatment | 36 |
| 5582STDY7759939 | 31/10/2017 | Kisu | Intensive | Damba | ERS2983603 | pre_treatment | 37 |
| 5582STDY7759930 | 05/12/2017 | Kisu | Intensive | Damba | ERS2983595 | post_treatment | 38 |
| 5582STDY7759954 | 06/12/2017 | Kisu | Intensive | Damba | ERS2983615 | post_treatment | 38 |
| 5582STDY7770954 | 06/12/2017 | Kisu | Intensive | Damba | ERS3016713 | post_treatment | 39 |
| 5582STDY7770961 | 05/12/2017 | Kisu | Intensive | Damba | ERS3016720 | post_treatment | 39 |
| 5582STDY7759962 | 06/12/2017 | Kisu | Intensive | Damba | ERS2983621 | post_treatment | 39 |
| 5582STDY7770943 | 24/08/2017 | Zingoola | Standard | Koome | ERS3016702 | pre_treatment | 51 |
| 5582STDY7770986 | 24/08/2017 | Zingoola | Standard | Koome | ERS3016745 | pre_treatment | 51 |
| 5582STDY7759964 | 22/08/2017 | Zingoola | Standard | Koome | ERS2983623 | pre_treatment | 51 |
| 5582STDY7759965 | 26/09/2017 | Zingoola | Standard | Koome | ERS2983624 | post_treatment | 51 |
| 5582STDY7770951 | 24/08/2017 | Zingoola | Standard | Koome | ERS3016710 | pre_treatment | 52 |
| 5582STDY7770978 | 24/08/2017 | Zingoola | Standard | Koome | ERS3016737 | pre_treatment | 52 |
| 5582STDY7771067 | 22/08/2017 | Zingoola | Standard | Koome | ERS3016793 | pre_treatment | 53 |
| 5582STDY7759887 | 25/10/2017 | Kitosi | Intensive | Koome | ERS2983565 | pre_treatment | 89 |
| 5582STDY7759966 | 26/10/2017 | Kitosi | Intensive | Koome | ERS2983625 | pre_treatment | 89 |
| 5582STDY7759919 | 28/11/2017 | Kitosi | Intensive | Koome | ERS2983586 | post_treatment | 89 |
| 5582STDY7771000 | 19/10/2017 | Busi | Intensive | Koome | ERS3016752 | pre_treatment | 5 |
| 5582STDY7724238 | 08/08/2017 | Kachanga | Standard | Damba | ERS2891500 | pre_treatment | 80 |
| 5582STDY7724230 | 12/09/2017 | Kachanga | Standard | Damba | ERS2891492 | post_treatment | 80 |
| 5582STDY7724241 | 18/10/2017 | Busi | Intensive | Koome | ERS2891503 | pre_treatment | 6 |
| 5582STDY7724316 | 17/10/2017 | Busi | Intensive | Koome | ERS2891578 | pre_treatment | 7 |
| 5582STDY7771044 | 24/08/2017 | Zingoola | Standard | Koome | ERS3016785 | pre_treatment | 54 |
| 5582STDY7771075 | 22/08/2017 | Zingoola | Standard | Koome | ERS3016796 | pre_treatment | 54 |
| 5582STDY7724294 | 23/08/2017 | Zingoola | Standard | Koome | ERS2891556 | pre_treatment | 54 |
| 5582STDY7771002 | 24/08/2017 | Zingoola | Standard | Koome | ERS3016754 | pre_treatment | 55 |
| 5582STDY7759974 | 25/10/2017 | Kitosi | Intensive | Koome | ERS2983631 | pre_treatment | 90 |
| 5582STDY7770971 | 11/10/2017 | Kakeeka | Standard | Damba | ERS3016730 | pre_treatment | 28 |
| 5582STDY7724249 | 10/10/2017 | Kakeeka | Standard | Damba | ERS2891511 | pre_treatment | 28 |
| 5582STDY7770924 | 12/10/2017 | Kakeeka | Standard | Damba | ERS3016683 | pre_treatment | 29 |
| 5582STDY7724266 | 11/10/2017 | Kakeeka | Standard | Damba | ERS2891528 | pre_treatment | 29 |
| 5582STDY7724247 | 12/10/2017 | Kakeeka | Standard | Damba | ERS2891509 | pre_treatment | 29 |
| 5582STDY7724248 | 15/11/2017 | Kakeeka | Standard | Damba | ERS2891510 | post_treatment | 30 |
| 5582STDY7771014 | 10/10/2017 | Kakeeka | Standard | Damba | ERS3016773 | pre_treatment | 30 |
| 5582STDY7724301 | 10/08/2017 | Kachanga | Standard | Damba | ERS2891563 | pre_treatment | 81 |
| 5582STDY7771076 | 24/10/2017 | Kitosi | Intensive | Koome | ERS3016797 | pre_treatment | 91 |
| 5582STDY7770977 | 15/07/2017 | Katooke | Intensive | Damba | ERS3016736 | pre_treatment | 70 |
| 5582STDY7771011 | 23/08/2017 | Zingoola | Standard | Koome | ERS3016770 | pre_treatment | 56 |
| 5582STDY7770919 | 23/08/2017 | Zingoola | Standard | Koome | ERS3016678 | pre_treatment | 56 |
| 5582STDY7771091 | 22/08/2017 | Zingoola | Standard | Koome | ERS3016802 | pre_treatment | 57 |
| 5582STDY7724310 | 23/08/2017 | Zingoola | Standard | Koome | ERS2891572 | pre_treatment | 57 |
| 5582STDY7724272 | 07/11/2017 | Lugumba | Standard | Lugumba | ERS2891534 | pre_treatment | 95 |
| 5582STDY7724264 | 13/12/2017 | Lugumba | Standard | Lugumba | ERS2891526 | post_treatment | 95 |
| 5582STDY7770969 | 12/07/2017 | Katooke | Intensive | Damba | ERS3016728 | pre_treatment | 71 |
| 5582STDY7770979 | 12/10/2017 | Kakeeka | Standard | Damba | ERS3016738 | pre_treatment | 31 |
| 5582STDY7759883 | 06/12/2017 | Kisu | Intensive | Damba | ERS2983561 | post_treatment | 40 |
| 5582STDY7759900 | 30/10/2017 | Kisu | Intensive | Damba | ERS2983569 | pre_treatment | 40 |
| 5582STDY7759955 | 01/11/2017 | Kisu | Intensive | Damba | ERS2983616 | pre_treatment | 40 |
| 5582STDY7770922 | 06/12/2017 | Kisu | Intensive | Damba | ERS3016681 | post_treatment | 40 |
| 5582STDY7759904 | 17/08/2017 | Katooke | Intensive | Damba | ERS2983573 | post_treatment | 72 |
| 5582STDY7759916 | 01/11/2017 | Kisu | Intensive | Damba | ERS2983583 | pre_treatment | 41 |
| 5582STDY7724287 | 09/10/2017 | Kakeeka | Standard | Damba | ERS2891549 | pre_treatment | 32 |
| 5582STDY7724273 | 10/10/2017 | Kakeeka | Standard | Damba | ERS2891535 | pre_treatment | 33 |
| 5582STDY7724236 | 23/11/2017 | Busi | Intensive | Koome | ERS2891498 | post_treatment | 8 |
| 5582STDY7724244 | 22/11/2017 | Busi | Intensive | Koome | ERS2891506 | post_treatment | 8 |
| 5582STDY7724253 | 18/10/2017 | Busi | Intensive | Koome | ERS2891515 | pre_treatment | 8 |
| 5582STDY7724291 | 17/10/2017 | Busi | Intensive | Koome | ERS2891553 | pre_treatment | 8 |
| 5582STDY7770990 | 23/11/2017 | Busi | Intensive | Koome | ERS3016763 | post_treatment | 9 |
| 5582STDY7724315 | 23/11/2017 | Busi | Intensive | Koome | ERS2891577 | post_treatment | 9 |
| 5582STDY7771068 | 23/10/2017 | Kitosi | Intensive | Koome | ERS3016794 | pre_treatment | 92 |
| 5582STDY7724278 | 22/08/2017 | Zingoola | Standard | Koome | ERS2891540 | pre_treatment | 58 |
| 5582STDY7759941 | 27/09/2017 | Zingoola | Standard | Koome | ERS2983605 | post_treatment | 59 |
| 5582STDY7771036 | 24/08/2017 | Zingoola | Standard | Koome | ERS3016782 | pre_treatment | 60 |
| 5582STDY7771051 | 23/08/2017 | Zingoola | Standard | Koome | ERS3016787 | pre_treatment | 60 |
| 5582STDY7771007 | 18/10/2017 | Busi | Intensive | Koome | ERS3016759 | pre_treatment | 10 |
| 5582STDY7724308 | 18/10/2017 | Busi | Intensive | Koome | ERS2891570 | pre_treatment | 11 |
| 5582STDY7724276 | 22/11/2017 | Busi | Intensive | Koome | ERS2891538 | post_treatment | 11 |
| 5582STDY7759925 | 27/09/2017 | Zingoola | Standard | Koome | ERS2983591 | post_treatment | 61 |
| 5582STDY7759918 | 23/10/2017 | Kitosi | Intensive | Koome | ERS2983585 | pre_treatment | 93 |
| 5582STDY7759926 | 24/10/2017 | Kitosi | Intensive | Koome | ERS2983592 | pre_treatment | 93 |
| 5582STDY7724229 | 18/10/2017 | Busi | Intensive | Koome | ERS2891491 | pre_treatment | 12 |
| 5582STDY7724227 | 17/10/2017 | Busi | Intensive | Koome | ERS2891489 | pre_treatment | 13 |
| 5582STDY7724235 | 19/10/2017 | Busi | Intensive | Koome | ERS2891497 | pre_treatment | 13 |
| 5582STDY7724268 | 22/11/2017 | Busi | Intensive | Koome | ERS2891530 | post_treatment | 13 |
| 5582STDY7724300 | 23/11/2017 | Busi | Intensive | Koome | ERS2891562 | post_treatment | 13 |
| 5582STDY7770959 | 24/08/2017 | Zingoola | Standard | Koome | ERS3016718 | pre_treatment | 62 |
| 5582STDY7771059 | 22/08/2017 | Zingoola | Standard | Koome | ERS3016790 | pre_treatment | 62 |
| 5582STDY7771010 | 24/08/2017 | Zingoola | Standard | Koome | ERS3016769 | pre_treatment | 63 |
| 5582STDY7770929 | 27/09/2017 | Zingoola | Standard | Koome | ERS3016688 | post_treatment | 64 |
| 5582STDY7770935 | 24/08/2017 | Zingoola | Standard | Koome | ERS3016694 | pre_treatment | 64 |
| 5582STDY7724302 | 23/08/2017 | Zingoola | Standard | Koome | ERS2891564 | pre_treatment | 64 |
| 5582STDY7771028 | 24/08/2017 | Zingoola | Standard | Koome | ERS3016779 | pre_treatment | 64 |
| 5582STDY7759888 | 13/07/2017 | Katooke | Intensive | Damba | ERS2983566 | pre_treatment | 73 |
| 5582STDY7759928 | 16/08/2017 | Katooke | Intensive | Damba | ERS2983594 | post_treatment | 73 |
| 5582STDY7770944 | 13/09/2017 | Kachanga | Standard | Damba | ERS3016703 | post_treatment | 82 |
| 5582STDY7759936 | 15/08/2017 | Katooke | Intensive | Damba | ERS2983601 | pre_treatment | 74 |
| 5582STDY7759944 | 13/09/2017 | Kachanga | Standard | Damba | ERS2983608 | post_treatment | 82 |
| 5582STDY7770976 | 18/10/2017 | Busi | Intensive | Koome | ERS3016735 | pre_treatment | 14 |
| 5582STDY7770953 | 24/08/2017 | Zingoola | Standard | Koome | ERS3016712 | pre_treatment | 65 |
| 5582STDY7759894 | 28/09/2017 | Zingoola | Standard | Koome | ERS2983557 | post_treatment | 66 |
| 5582STDY7759901 | 28/09/2017 | Zingoola | Standard | Koome | ERS2983570 | post_treatment | 66 |
| 5582STDY7771008 | 17/10/2017 | Busi | Intensive | Koome | ERS3016760 | pre_treatment | 15 |
| 5582STDY7770982 | 07/08/2017 | Kachanga | Standard | Damba | ERS3016741 | pre_treatment | 83 |
| 5582STDY7770936 | 13/09/2017 | Kachanga | Standard | Damba | ERS3016695 | post_treatment | 84 |
| 5582STDY7770927 | 24/08/2017 | Zingoola | Standard | Koome | ERS3016686 | pre_treatment | 67 |
| 5582STDY7759933 | 26/09/2017 | Zingoola | Standard | Koome | ERS2983598 | post_treatment | 67 |
| 5582STDY7724286 | 23/08/2017 | Zingoola | Standard | Koome | ERS2891548 | pre_treatment | 67 |
| 5582STDY7770945 | 27/09/2017 | Zingoola | Standard | Koome | ERS3016704 | post_treatment | 67 |
| 5582STDY7770966 | 22/11/2017 | Busi | Intensive | Koome | ERS3016725 | post_treatment | 16 |
| 5582STDY7724245 | 19/10/2017 | Busi | Intensive | Koome | ERS2891507 | pre_treatment | 16 |
| 5582STDY7724313 | 18/10/2017 | Busi | Intensive | Koome | ERS2891575 | pre_treatment | 16 |
| 5582STDY7724237 | 18/10/2017 | Busi | Intensive | Koome | ERS2891499 | pre_treatment | 16 |
| 5582STDY7724304 | 08/11/2017 | Lugumba | Standard | Lugumba | ERS2891566 | pre_treatment | 96 |
| 5582STDY7770983 | 17/10/2017 | Busi | Intensive | Koome | ERS3016742 | pre_treatment | 17 |
| 5582STDY7759938 | 05/12/2017 | Kisu | Intensive | Damba | ERS2983602 | post_treatment | 42 |
| 5582STDY7771053 | 01/11/2017 | Kisu | Intensive | Damba | ERS3016789 | pre_treatment | 43 |
| 5582STDY7771061 | 31/10/2017 | Kisu | Intensive | Damba | ERS3016792 | pre_treatment | 43 |
| 5582STDY7759906 | 31/10/2017 | Kisu | Intensive | Damba | ERS2983574 | pre_treatment | 43 |
| 5582STDY7759898 | 07/12/2017 | Kisu | Intensive | Damba | ERS2983567 | post_treatment | 44 |
| 5582STDY7759882 | 06/12/2017 | Kisu | Intensive | Damba | ERS2983560 | post_treatment | 44 |
| 5582STDY7724317 | 13/09/2017 | Kachanga | Standard | Damba | ERS2891579 | post_treatment | 85 |
| 5582STDY7724263 | 11/10/2017 | Kakeeka | Standard | Damba | ERS2891525 | pre_treatment | 34 |
| 5582STDY7724279 | 10/10/2017 | Kakeeka | Standard | Damba | ERS2891541 | pre_treatment | 34 |
| 5582STDY7770916 | 11/10/2017 | Kakeeka | Standard | Damba | ERS3016675 | pre_treatment | 34 |
| 5582STDY7724271 | 09/10/2017 | Kakeeka | Standard | Damba | ERS2891533 | pre_treatment | 34 |
| 5582STDY7770957 | 07/08/2017 | Kachanga | Standard | Damba | ERS3016716 | pre_treatment | 86 |
| 5582STDY7770973 | 09/08/2017 | Kachanga | Standard | Damba | ERS3016732 | pre_treatment | 86 |
| 5582STDY7770950 | 10/10/2017 | Kakeeka | Standard | Damba | ERS3016709 | pre_treatment | 35 |
| 5582STDY7771029 | 01/11/2017 | Kisu | Intensive | Damba | ERS3016780 | pre_treatment | 45 |
| 5582STDY7770975 | 17/10/2017 | Busi | Intensive | Koome | ERS3016734 | pre_treatment | 18 |
| 5582STDY7770984 | 19/10/2017 | Busi | Intensive | Koome | ERS3016743 | pre_treatment | 19 |
