# Supplementary material for: Genome-wide analysis of Schistosoma mansoni reveals limited population structure and possible praziquantel drug selection pressure within Ugandan hot-spot communities
Source: PLoS Negl Trop Dis. 2022 Aug 18;16(8):e0010188. doi: 10.1371/journal.pntd.0010188 (PMC9426917; doi:10.1371/journal.pntd.0010188)
Supplement: S2 Table — (region X on Fig 4B). (DOCX) [file pntd.0010188.s006.docx]

**S2 Table.** Protein coding genes present in the region of highest genetic differentiation on chromosome 5. (region X on Fig. 4B).

**Chromosome GeneID Function_name**

SM_V7_5 Smp_026090 Ras-related GTP-binding protein D

SM_V7_5 Smp_026160 Growth hormone-inducible transmembrane protein

SM_V7_5 Smp_026190 Probable U3 small nucleolar RNA-associated protein 11

SM_V7_5 Smp_101230 Phenylalanine--tRNA ligase alpha subunit

SM_V7_5 Smp_102040 Guanine nucleotide-binding protein subunit beta-2-like 1

SM_V7_5 Smp_129950 RNA-binding protein 12

SM_V7_5 Smp_129960 Nestin

SM_V7_5 Smp_129970 Hypothetical protein

SM_V7_5 Smp_136240 Vesicle-associated membrane protein/synaptobrevin-binding protein

SM_V7_5 Smp_136260 Glutamine--fructose-6-phosphate aminotransferase [isomerizing] 2

SM_V7_5 Smp_136280 Regulator of telomere elongation helicase 1

SM_V7_5 Smp_136300 tyrosine kinase, TK group, Src family

SM_V7_5 Smp_136310 Sodium/bile acid cotransporter

SM_V7_5 Smp_178810 26S proteasome non-ATPase regulatory subunit 13

SM_V7_5 Smp_242830 Prolyl 3-hydroxylase OGFOD1

SM_V7_5 Smp_242860 Trafficking protein particle complex subunit 8

SM_V7_5 Smp_247640 ATPase synthesis protein 25, mitochondrial

SM_V7_5 Smp_247650 Eukaryotic translation initiation factor 3 subunit D

SM_V7_5 Smp_267060 Tether containing UBX domain for GLUT4

SM_V7_5 Smp_314360 Phenylalanine--tRNA ligase alpha subunit

SM_V7_5 Smp_316680 Phenylalanine--tRNA ligase alpha subunit

SM_V7_5 Smp_332100 40S ribosomal protein S28

SM_V7_5 Smp_341690 Golgi-specific brefeldin A-resistance guanine nucleotide exchange factor 1

SM_V7_5 Smp_346850 Acetyl-coenzyme A carboxylase carboxyl transferase subunit beta, chloroplastic

SM_V7_5 Smp_347070 Calcium/calmodulin-dependent protein kinase type IV
