# Supplementary material for: Genome-wide analysis of Schistosoma mansoni reveals limited population structure and possible praziquantel drug selection pressure within Ugandan hot-spot communities
Source: PLoS Negl Trop Dis. 2022 Aug 18;16(8):e0010188. doi: 10.1371/journal.pntd.0010188 (PMC9426917; doi:10.1371/journal.pntd.0010188)
Supplement: S3 Table — Genes are from GO hierarchies for Molecular Function (MF), Biological Processes (BP), KEGG pathways and Cellular Component (CC) within the post-treatment (Tp2). Standard (Std) and Intensive (Int) groups with respective adjusted p-values (Padj). Asterisks mark terms that remain enriched in autosomal gene sets. (DOCX) [file pntd.0010188.s007.docx]

**S3 Table.** GO terms significantly over-represented among genes overlapping regions of different natural selection. Genes are from GO hierarchies for Molecular Function (MF), Biological Processes (BP), KEGG pathways and Cellular Component (CC) within the post-treatment (Tp2). Standard (Std) and Intensive (Int) groups with respective adjusted p-values (P_adj_). Asterisks mark terms that remain enriched in autosomal gene sets.

| **Group** | **GO** | **Term_name** | **GO_ID** | **number of genes** | **P_adj_** |
| --- | --- | --- | --- | --- | --- |
| Tp2 | MF | Purine-nucleoside phosphorylase activity | [GO:0004731](https://biit.cs.ut.ee/gprofiler/convert?organism=scmansprjea36577&query=GO:0004731) | 5 | 1.616×10^-8^ |
| Tp2 | MF | Transferase activity, transferring pentosyl groups | [GO:0016763](https://biit.cs.ut.ee/gprofiler/convert?organism=scmansprjea36577&query=GO:0016763) | 6 | 1.352×10^-5^ |
| Tp2 | MF | Transferase activity, transferring glycosyl groups | GO:0016757 | 10 | 8.088×10^-4^ |
| Tp2 | MF | S-methyl-5-thioadenosine phosphorylase activity | GO:0017061 | 2 | 1.678×10^-2^ |
| Tp2 | MF | Phosphatidylinositol-4,5-bisphosphate 4-phosphatase activity | GO:0034597 | 2 | 1.678×10^-2^ |
| Tp2 | MF | Phosphatidylinositol-4,5-bisphosphate phosphatase activity | GO:0106019 | 2 | 1.678×10^-2^ |
| Tp2 | MF | Phosphatidylinositol bisphosphate phosphatase activity | GO:0034593 | 2 | 4.999×10^-2^ |
| Tp2 | MF | Phosphatidylinositol phosphate 4-phosphatase activity | GO:0034596 | 2 | 4.999×10^-2^ |
| Tp2 | BP | Nucleoside metabolic process | GO:0009116 | 5 | 8.312×10^-3^ |
| Tp2 | BP | Glycosyl compound metabolic process | GO:1901657 | 5 | 8.312×10^-3^ |
| Tp2 | BP | Carbohydrate derivative metabolic process | GO:1901135 | 11 | 9.720×10^-3^ |
| Tp2 | BP | L-methionine salvage from methylthioadenosine | GO:0019509 | 2 | 4.958×10^-2^ |
| Tp2 | BP | Amino acid salvage | GO:0043102 | 2 | 4.958×10^-2^ |
| Tp2 | BP | L-methionine biosynthetic process | GO:0071265 | 2 | 4.958×10^-2^ |
| Tp2 | BP | L-methionine salvage | GO:0071267 | 2 | 4.958×10^-2^ |
| Tp2 | CC | Late endosome membrane | GO:0031902 | 2 | 4.997X10^-2^ |
| Tp2 | KEGG | Fatty acid Metabolism | KEGG:01212 | 3 | 1.363X10^-2^ |
| Int | MF | Peroxidase activity* | GO:0004601 | 5 | 2.809×10^-4^ |
| Int | MF | Oxidoreductase activity, acting on peroxide as acceptor* | GO:0016684 | 5 | 4.164×10^-4^ |
| Int | MF | Antioxidant activity* | GO:0016209 | 5 | 2.627×10^-3^ |
| Int | MF | Heme binding* | GO:0020037 | 5 | 4.241×10^-3^ |
| Int | MF | Tetrapyrrole binding* | GO:0046906 | 5 | 4.241×10^-3^ |
| Int | BP | Response to toxic substance* | GO:0009636 | 5 | 1.658X10^-3^ |
| Int | BP | Cellular response to toxic substance* | GO:0097237 | 5 | 1.658X10^-3^ |
| Int | BP | Detoxification* | GO:0098754 | 5 | 1.658X10^-3^ |
| Int | BP | Cellular oxidant detoxification* | GO:0098869 | 5 | 1.658X10^-3^ |
| Int | BP | Cellular detoxification* | GO:1990748 | 5 | 1.658X10^-3^ |
| Std | MF | tRNA-specific adenosine deaminase activity* | GO:0008251 | 2 | 8.869X10^-3^ |
| Std | MF | tRNA-specific adenosine-34 deaminase activity* | GO:0052717 | 2 | 8.369X10^-3^ |
| Std | MF | adenosine deaminase activity* | GO:0004000 | 2 | 4.981X10^-2^ |
| Std | CC | tRNA-specific adenosine-34 deaminase complex* | GO:0052718 | 2 | 5.060X10^-2^ |
